# Observing ageism implicitly using the numerical parity judgment task

**DOI:** 10.1038/s41598-023-40876-1

**Published:** 2023-12-01

**Authors:** D. Aisenberg-Shafran, A. Henik, N. Gronau

**Affiliations:** 1 Department of Clinical Psychology of Adulthood and Aging, Ruppin Academic Center, 4025000 Emek Hefer, Israel; 2 Department of Psychology and Zlotowski Center for Neuroscience, Ben-Gurion University of the Negev, Beer-Sheva, Israel; 3 Department of Psychology, The Open University, Raanana, Israel

**Keywords:** Psychology, Human behaviour

## Abstract

Objective magnitude representations may be prone to subjective percepts when judging human beings. An elderly man is clearly “large” in terms of age. But, is he truly perceived as “big” in our minds? We investigated whether “objective” representation of age interacts with subjective stereotypical percepts of aging, using a numeral classification task preceded by prime images containing human figures. First, prime images of children and young adults demonstrated a positive correlation between perceived age and numerical size. Second, negatively and positively valenced prime images were associated with small and big numerical values, respectively. Third, joint effects of age and valence on numerical value perception revealed a linkage between old adults and small numerical values. It seems that magnitude perception is vulnerable to implicit subjective biases and stereotypical judgments dominate objective magnitude representation.

## Introduction

The representation of old adults has been shown to comprise both positive and negative percepts^1^. Typically, when positive perceptions are observed, they are related to kindness, calmness, and wisdom (e.g.,^2^). When negative perceptions are observed, they are related to disability, sickness, and dysfunction (e.g.,^3^) and are often termed “ageism”. Ageing stereotypes may be observed explicitly, yet, since these are often negative percepts, they may be suppressed or silenced. In the current study, we designed an implicit task to explore negative age stereotypes through the assessment of indirect age-number associations. Humans possess a core numerical system that allows us to perceive, manipulate, and compare discrete quantities (e.g.,^4–7^). Much research has been devoted to studying the mechanisms underlying number processing, along with their relations to other magnitude representations of size, space and time (e.g.,^8,9^). Therefore, we suggest an interdisciplinary investigation of the interaction between numerical magnitude perception and social concepts laden with emotional valence.

Numerical processing has been associated with a variety of magnitude representations, among these, stimuli size has been the focus of ample research. Henik and Tzelgov^10^ demonstrated that numbers—discrete symbols denoting magnitude or quantity—interact with physical size—a non-symbolic value representing continuous magnitudes. For instance, in the well-known *size congruity effect* (SiCE), responses to physically large digits were shown to be faster when digits denoted large numerical values than when they denoted small numerical values (e.g.,^10^; see also^11–14^). Further research has shown that perceived size, not necessarily physical size, may play a critical role in size-number interaction effects^15^. Other studies using images of real-world stimuli (rather than symbolic digits) have highlighted the importance of more abstract, semantic knowledge concerning an object’s real-world size, regardless of its retinal or its perceived size. Thus, for instance, in a parity judgment task performed on digit targets, a prime image of a conceptually small (snail) or a large (horse) animal was found to facilitate responses to a small or a large target digit, respectively^16^ (see also^17^). Priming occurred despite the presentation of the animal images at a fixed retinal size and despite the fact that the prime stimuli were strictly irrelevant to task requirements, suggesting that extraction of conceptual size knowledge is largely automatic. While the relation between retinal size and conceptual (i.e., real-world) size has been long demonstrated^18^ (see also^19,20^), the direct associative relations between conceptual size and numerical value have been rarely documented (e.g.,^16^).

Size is a core property of objects (e.g.,^21^), however a conceptual size representation may be influenced not only by objective size parameters but also by more subjective percepts that may affect one’s judgment of an object’s physical dimensions. Indeed, several studies have shown that positively valued or desired stimuli (e.g., a coin judged by children from a low socio-economic status) were rated as larger than emotionally neutral objects (e.g.,^22,23^). Subjective percepts may be particularly dominant when judging human beings, since implicit attitudes, stereotypes and emotions may interact with more objective knowledge about one’s physical dimensions (e.g., height) or, relatedly and perhaps more relevant to a person’s identity—his/her age (e.g., classifying one as a child, an adult or an old adult). Consider, for instance, an old male adult–he is clearly “large” in terms of his age and his lifetime experience. He is also physically bigger (e.g., taller, wider) than a child. But, is he truly perceived as “big” or “large” in our minds? There are reasons to believe that percepts associated with aging and with old age, such as weakness or vulnerability^1,3^, interact with more objective age estimations, yielding a reduced or a diminished representation of old adults, compared to young adults. That is, negative valence associated with stereotypes of elders and presumably valued as low (on an implicit social/emotional scale) may interfere and/or compete with an objective representation of an elderly person’s age. Indeed, priming young participants with different concepts of “elderly” has been shown to impede (e.g., slow down) their performance across a wide variety of tasks (e.g.,^24–26^).

In the present study, we examined whether a subjective, stereotypical percept associated with aging does in fact interact with a more objective representation of human age. By using a priming paradigm, in which images of children and of young adults served as primes in a numeral-target classification task, we assessed the associative relations between “objective” age perception and numerical value (Experiment 1). We hypothesized that similar to conceptual size findings (e.g.,^16^), age would be positively associated with numerical value. We further employed a similar paradigm, in which emotionally-laden images served as prime stimuli, to examine the effects of “subjective” attitudes (elicited by positive and negative valence images) on number perception (Experiment 2). Here, our hypothesis was that negative images would be associated with small numbers, and vice versa. Finally, the potential interaction between age and valence was tested in an experiment involving prime images of children, young adults *and* old adults. We posited that when subjective stereotypical judgments compete with objective age representation, the former would dominate the latter, yielding a significantly reduced, or a lessened representation of old adults, compared to the young adults. Consequently, elders would be more strongly associated with small than with large numerical values (Experiment 3).

For all the experiments described herein, ethical approval was obtained from the behavioral ethics board of Ben-Gurion University and informed consent was provided by all participants. All methods were performed in accordance with the relevant guidelines and regulations and data does not contain any identifying information/images.

### Experiment 1–age as an objective magnitude parameter

As explained above, at the first stage we wished to examine how the objective parameter of age is associated with numerical value. To this end, we used a priming paradigm, similar to the one employed by Gabay et al.^16^, in which images of young children or young adults (but not old adults) were presented prior to the appearance of target digits. Two experiments were conducted, in which participants performed two different tasks on the target digit stimuli. In Experiment 1A, participants explicitly responded to the digit’s numerical value (“is the number bigger or smaller than 5”), allowing a direct assessment of the associative relations between the two types of magnitudes (age, numbers). In Experiment 1B, the indirect effects of perceived age on numerical magnitude were assessed, by using a parity judgment task (“is the number odd or even”), which is orthogonal to the digit’s value or magnitude. We hypothesized that perceived age is associated with numerical size, such that an image of a young child would prime digits denoting small numerical values, while an image of a young adult would prime digits denoting big numerical values, regardless of the task instructions. Importantly, by using an indirect task that does not require an explicit judgment of size magnitude (Experiment 1B), one could rule out response bias as a possible account for any potential priming effect. Consequently, a stronger argument for the associative relations between perceived age and numerical value could be made. Note that perceived age is strongly correlated with perceived (or conceived) size: young adults are both older and physically bigger (e.g., taller, wider) than children. While we refer to both types of magnitudes (i.e., age, size) as “objective” size parameters, our focus in this as well as in the following studies is on humans’ age representation. We will discuss the potential implications of this confound (among the two magnitude parameters) in Experiment 3.

## Materials and method

### Participants

Eighteen undergraduate students (mean age = 23.9 years, SD = 2.4, range = 19–28) participated in Experiment 1A, and an additional 18 students (mean age = 23.5 years, SD = 1.98, range = 20–27) participated in Experiment 1B. All participants were native Hebrew speakers, right-handed, with normal or corrected-to-normal visual acuity, that were naïve with regard to the purpose of the experiment. The number of participants per experiment followed a statistical power analysis using G*Power^27^ see note 1 in Appendix B.

### Stimuli

Prime pictures were selected from the International Affective Picture System (IAPS)^28^. Six images presenting an individual male young child and six images presenting an individual male young adult were chosen. Based on a pilot survey conducted among eight independent raters, the children within the “young” age category were estimated to be between the ages of 1- and 12-years old (mean = 5.9, SD = 1.1), while the young adults within the “adult” age category were estimated to be between the ages of 22- and 49-years-old (mean = 32.7, SD = 2.5). The images from the two age categories were matched for their valence and arousal parameters (provided by the IAPS database), as both categories possessed intermediate scores for these parameters (mean valence: children—5.0, SD = 1.5, young adults—5.1, SD = 1.3; mean arousal: children—3.7, SD = 1.9, young adults—3.6, SD = 2, see TableA.1 in Appendix A). In addition, special care was taken to match images from the two categories for other parameters, such as race and the degree of zoom-in of the photographed figures (i.e., whether images contained a person’s face only or a whole body). All images spanned 4˚ by 3˚, from a viewing distance of approximately 60 cm. Target stimuli consisted of digits (approximately 1.8˚ * 3.2˚) that either depicted small (1, 2) or big (8, 9) numerical values. Each of the six prime images within the two age conditions was presented with each of the two digits within the two number conditions, and each of these combinations was repeated three times in the course of the block, yielding a total of 144 trials per block. Participants conducted 16 practice trials before performing the two experimental blocks (yielding an overall total of 288 experimental trials).

### Procedure

**Figure 1:**
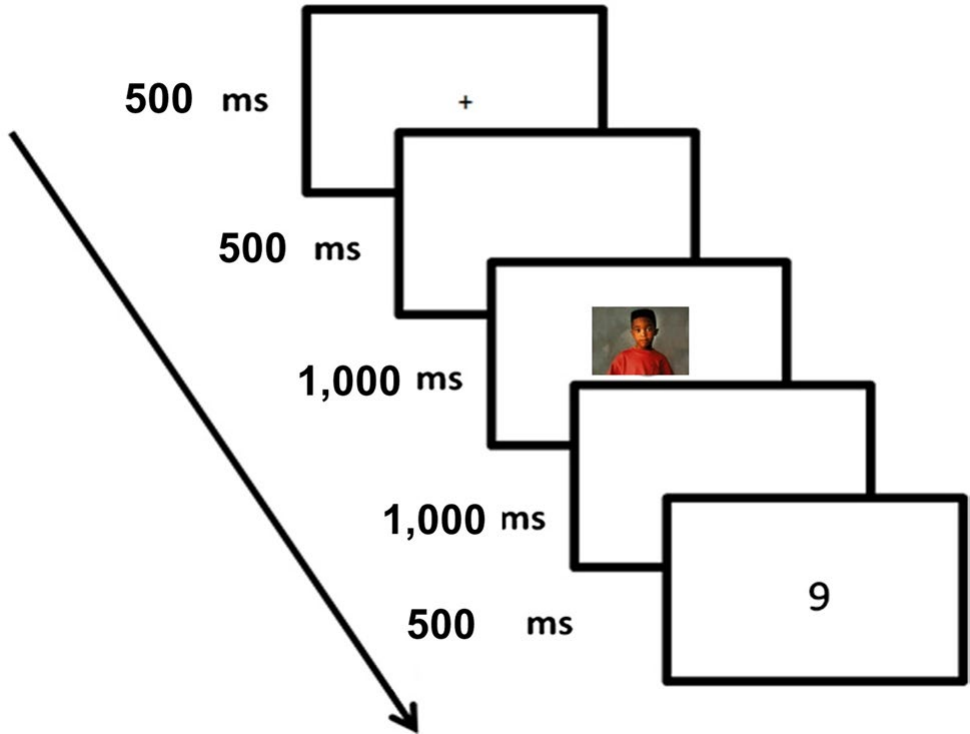
Trial sequence in Experiments 1A and 1B.

Participants were tested in a dimly-illuminated room. They were instructed to maintain fixation at the center of the screen throughout the experiment. Each trial began with the appearance of a central fixation cross (0.8˚) for 500 ms. After a blank interval of 500 ms, a prime image appeared for 1000 ms. Participants were instructed to respond vocally to the prime picture by indicating whether the person in the image was “Young” (i.e., a child) or an “Adult” see note 2 in Appendix B. This was a unique request, to make sure they noticed the prime image. A microphone placed to the left of the participants collected their responses. After the appearance of the prime stimulus, there was an additional blank interval of 1000 ms, followed by a target digit presented for 500 ms (see Fig. 1 for a complete trial sequence). Participants were instructed to respond manually as fast as possible to the target’s identity, while maintaining accuracy. In Experiment 1A, they were asked to rapidly determine whether the target’s value was bigger or smaller than the number 5. Response mapping was counterbalanced such that half of the participants were asked to press the “L” key for a digit which was bigger than 5 and the “D” key for a digit which was smaller than 5, and vice versa. In Experiment 1B, participants were instructed to make a parity judgment and respond as fast as possible as to whether the target’s value was odd or even. Here again, responses were counterbalanced (e.g., press “L” for an even digit and “D” for an odd digit, and vice versa). A blank screen appeared at the end of each trial for 1000 ms.

### Results and discussion of experiment 1

Before analyzing the results (in Experiment 1A and 1B), RT (reaction time) of various mappings (for example- right/left hand responses; SNARC) was tested to avoid any response bias. No SNARC (spatial-numerical association of response codes) effect was found^17^, and no bias was found for mapping of priming conditions and target stimulus conditions. The lack of SNARC effect may seem surprising, but in line with Zebian^29^, SNARC effects often do not appear in studies with Hebrew speakers^30,31^, who read and write from right to left (opposite of reading numbers and Latin letters). In our study participants were Hebrew and Arabic native speakers, and hence their effect may be diminished.

**Figure 2:**
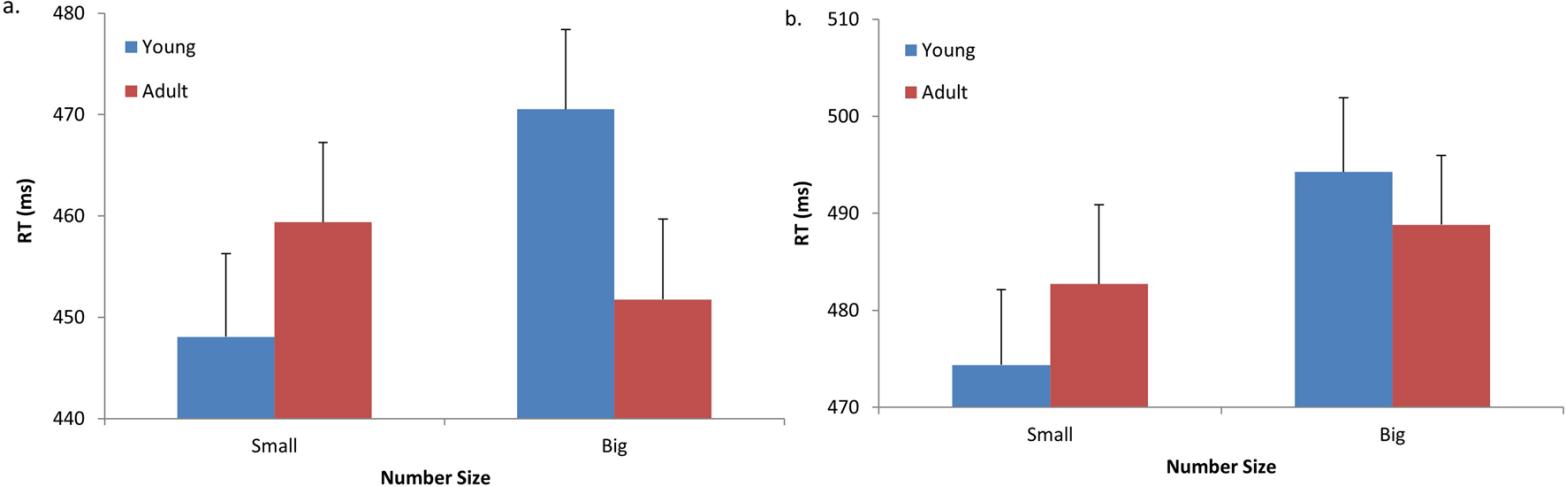
Mean reaction times (RT) to target digits, within the different conditions (Prime type, Number size) in Experiment 1. **a** Responses to the size-classification task (“bigger or smaller than 5”) in Experiment 1A; **b** Responses to the parity-judgment task (“odd or even”) in Experiment 1B. Error bars indicate the standard errors of the means.

Accuracy and mean RTs for correct responses were computed for each participant in each condition. Outlier responses (i.e., responses that were shorter than 150 ms, or were three standard deviations above/below the mean of each participant within each condition) were excluded from the analysis (0.3% in Experiment 1A, 1.7% in Experiment 1B). The overall error rate was low (0.02 in Experiment 1A, 0.05 in Experiment 1B), and thus errors were not analyzed. Figure 2 presents the mean RTs in the different conditions within the two experimental tasks.

A repeated-measures analysis of variance (ANOVA) was conducted with Prime type (young, adult) and Number size (small, big) as factors. In Experiment 1A, none of the main effects were significant (Prime type: F(1, 17) = 2.9, $\upeta_{{\text{p}}}^{2}$ = 0.14, MSE = 108, *p* > 0.1, ns; Number size: F(1, 17) = 2.03, $\upeta_{{\text{p}}}^{2}$ = 0.10, MSE = 625, *p* > 0.17, ns). Importantly, there was a significant two-way interaction between Prime type and Number size, F(1, 17) = 15.1, $\upeta_{{\text{p}}}^{2}$ = 0.47, MSE = 288, *p* > 0.001, showing the expected pattern of results: RT was faster when small numbers were preceded by pictures depicting young children than when they were preceded by pictures depicting adults, F(1, 17) = 5.38, MSE = 214, *p* < 0.033; and, similarly, RT was faster when big numbers were preceded by pictures of adults than when they were preceded by pictures of children, F(1, 17) = 19.32, MSE = 181, *p* < 0.0003.

In Experiment 1B, the main effect of Prime type was not significant, F(1, 17) < 1, yet the main effect of Number size was marginally significant, F(1, 17) = 4.23, $\upeta_{{\text{p}}}^{2}$= 0.2, MSE = 539, *p* > 0.056, suggesting longer overall latencies for big than for small numbers. In accord with the results of Experiment 1A, there was a significant interaction between Prime type and Number size, F(1, 17) = 4.66, $\upeta_{{\text{p}}}^{2}$ = 0.21, MSE = 216, *p* > 0.04. This interaction stemmed from a pattern of results similar to that of Experiment 1A, yet the simple main effects failed to reach statistical significance level (Prime type effect in small numbers: F(1, 17) = 2.46, MSE = 338, *p* > 0.13, ns; Prime type effect in big numbers: F(1, 17) = 1.7, MSE = 159, *p* > 0.21, ns). Obtaining an interaction effect in Experiment 1B provided a replication of the results of Experiment 1A (albeit the former was somewhat weaker in its magnitude than the latter), further emphasizing the associative relations of age and numerical value. Critically, since the parity judgment task was orthogonal to the numerical value of the target, the interaction obtained between the two factors could not be an outcome of a simple response bias, rather, it most likely occurred on an a more associative, or conceptual level.

### Experiment 2–valence as a subjective magnitude parameter

Experiment 1 established the associative relation between age and numerical value. In Experiment 2 we wished to examine the potential effects of perceived valence on numerical processing. The joint results of these two experiments would provide a baseline, or an anchor, for the interactive influence of valence and age on number perception, tested in Experiment 3. In order to address the question of subjective valence effects on numeral perception, we followed the design employed in Experiment 1, using both direct and indirect methods of assessment; relating to the magnitude dimension, one directly seeks magnitude judgment (smaller/bigger than 5), while the other is disregarding the magnitude dimension (odd/even is not related to size). As mentioned earlier, we focused on emotions presumably associated with stereotypes of old adults, therefore images depicting sadness, poorness and/or weakness (vs. happiness) were selected as prime stimuli. In accordance with findings from several previous studies (e.g.,^25,26,32^), we hypothesized that negative valence (i.e., images depicting poorness or sadness) would relate to small numerical values while positive valence would relate to big numerical values (see note 3 in Appendix B).

## Materials and method

### Participants

Eighteen undergraduate students (mean age = 23.6 years, SD = 2.03, range = 19–27), participated in Experiment 2A, and an additional 18 students (mean age = 23.44 years, SD = 2.61, range = 18–30) participated in Experiment 2B. Participants in both studies were right-handed, native Hebrew speakers, with normal or corrected-to-normal visual acuity, who did not participate in previous experiments. All were naïve regarding the purpose of the experiment.

### Stimuli

Ten prime pictures depicting a human figure were selected from the IAPS database^28^. All images differed from the ones used in Experiment 1. Five pictures representing positive scenes (mean valence = 7.4, SD = 1.4), and five pictures representing negative scenes (mean valence = 2.6, SD = 1.4) were chosen for the two valence categories. The positive-category images generally depicted happy/desired situations (e.g., a baby smiling, a person drinking a beer), while the negative-category images depicted sad/pitiful situations (e.g., a child crying, an unhealthy person). Images were matched for their arousal level, yielding intermediate average arousal levels in both categories (mean arousal level for positive images: 4.5, SD = 2.3; for negative images: 4.6, SD = 2.0, see Table A.2 in Appendix A). In addition, they were matched as much as possible for other parameters, such as the gender, age, race, and the degree of zoom-in of the photographed figures, as well as the general brightness of the images. Since the IAPS database does not provide information about the nature of the specific emotion evoked by the images (but only about its general valence), we conducted a pilot survey among eight independent raters to assure that the images chosen for the negative (i.e., low valence) category depicted sadness and/or weakness, rather than other types of negative emotions (such as threat or fear).

Each of the five prime images within the two valence conditions was presented with each of the two digits within the two number conditions, and each of these combinations was repeated twice during a block, yielding a total of 80 trials per block. Participants conducted 16 practice trials before performing the three experimental blocks (yielding an overall total of 240 experimental trials). All other parameters were identical to those in Experiment 1.

### Procedure

The procedure was identical to that of Experiment 1, with one exception: participants were instructed to respond vocally to the prime picture by indicating whether the image depicted a “positive” or a “negative” scene. Thereafter, they had to respond manually to the digit target, deciding as fast as possible whether it was bigger or smaller than 5 (Experiment 2A), or whether it was odd or even (Experiment 2B).

### Results and discussion of experiment 2

**Figure 3:**
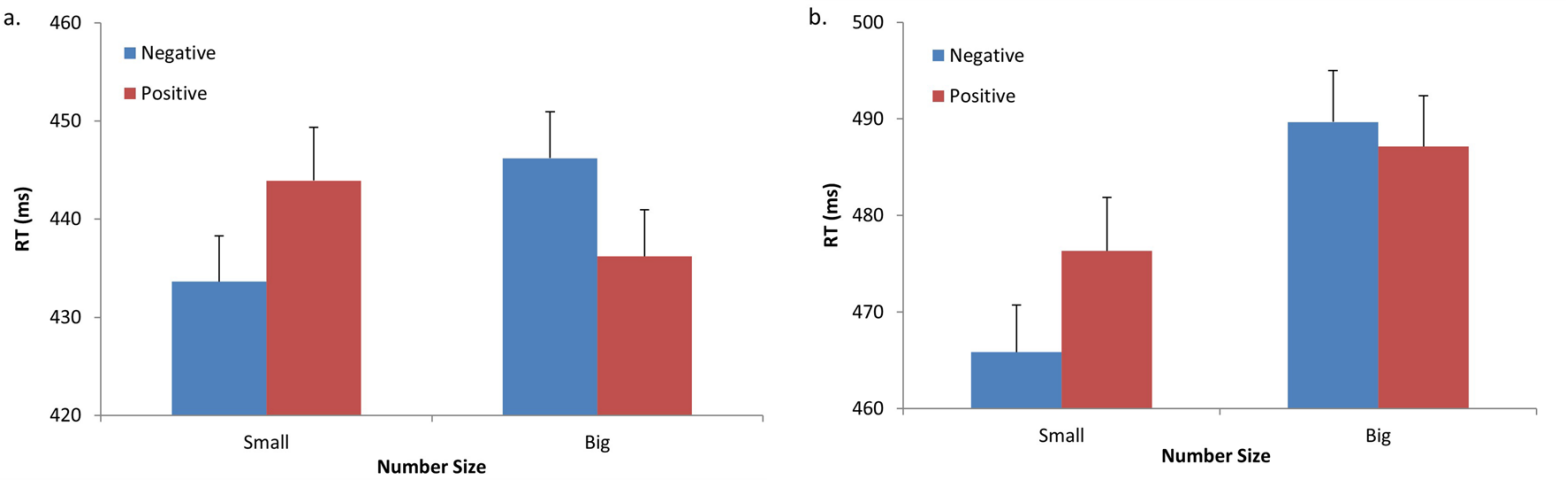
Mean reaction times (RT) to target digits, within the different conditions (Prime type, Number size) in Experiment 2. **a** Responses to the size-classification task (“bigger or smaller than 5”) in Experiment 2A; **b** Responses to the parity-judgment task (“odd or even”) in Experiment 2B. Error bars indicate the standard errors of the means.

Accuracy and mean RTs for correct responses were computed for each participant in each condition. Outlier responses (i.e., responses that were shorter than 150 ms or were three standard deviations above/below the mean of each participant within each condition) were excluded from the analysis (2% in Experiment 2A, 2.3% in Experiment 2B). The overall error rate was low (Experiment 2A: 0.03; Experiment 2B: 0.07), thus errors were not analyzed. Figure 3 presents the mean RTs in the different conditions within the two experimental tasks.

A repeated-measures ANOVA was conducted with Prime type (negative, positive) and Number size (small, big) as factors. In Experiment 2A, there were no significant main effects (all Fs < 1). Importantly, however, the two-way interaction between Prime type and Number size was statistically significant, F(1, 17) = 9.68, $\upeta_{{\text{p}}}^{2}$ = 0.36, MSE = 358, *p* < 0.006. As hypothesized, negative valence was associated with small numerical values and positive valence was associated with big numerical values: RT was significantly faster when negative pictures, compared to positive pictures, preceded small numbers, F(1, 17) = 7.67*,* MSE = 344*,* *p* < 0.013. In addition, RT was faster when positive pictures, compared to negative pictures, preceded big numbers (though this difference was only marginally significant, F(1, 17) = 3.3, MSE = 305, *p* < 0.08). In Experiment 2B, there was a significant main effect of Number size, F(1, 17) = 14.04, $\upeta_{{\text{p}}}^{2}$ = 0.45, MSE = 387, *p* < 0.005, indicating longer overall latencies for big than for small numbers, as observed in previous experiments. There was no main effect of Prime type (F < 1). As in Experiment 2A, the two-way interaction between Prime type and Number size was significant, F(1, 17) = 5.1, $\upeta_{{\text{p}}}^{2}$ = 0.23, MSE = 112, *p* < 0.05. Once again, RT was faster when small numbers were preceded by negative than by positive pictures, F(1, 17) = 3.36, MSE = 359, *p* < 0.05. The corresponding simple main effect, however, was non-significant among the big numerical values (F < 1).

Taken together, the results of Experiment 2A and 2B strongly suggest that negative valence depicting sadness and/or poorness is associated with small numerical values, while positive valence is associated with big numerical values (given equal arousal levels in both valence categories). These findings are in line with those of Holmes and Lourenco^33^, who suggested that the format of spatial organization extends from number representation also to emotional expression.

We now turn to the main goal of our research, namely, studying the potential interactive effects of age and valence on numerical size perception.

### Experiment 3–the interactive effects of age (objective magnitude) and valence (subjective magnitude) on numerical size perception

As explained earlier, despite the apparent linear relation between perceived age and number perception (according to which young ages are associated with small numerical values, while older ages are associated with big numerical values), we hypothesized that older adults who are conceived and stereotyped as weak, vulnerable and non-attractive would be associated with small rather than with big numerical values. The current experiment aimed to test this hypothesis by examining the priming effects of images of young children, young adults, and old adults on numerical targets. After establishing that age and valence affect numerical perception, even when they are strictly irrelevant to the task performed on the digit stimuli (e.g., parity judgment), we used an identical task, orthogonal to the digit’s value, in Experiment 3. Three levels of numeral values (Small, Medium, Big) were used in the parity judgment task, equivalent to the three prime age categories (note that the more direct size classification task—“bigger/smaller than 5”—uses two rather than three categories and was less appropriate for the present purpose). The numeral values used as targets were 1–2 (Small), 4–5 (Medium) and 8–9 (Big).

If “objective” age parameters dominate human representation, a linear trend should be observed, in which images of young children mainly prime small numbers, images of old adults mainly prime large numbers, and images of young adults fall somewhere in between these two categories, priming mainly intermediate numbers. If, however, social stereotypes and emotional valence factors overpower the effects of objective age judgment, old adults should be mainly associated with small rather than with big numbers. This may occur despite the use of a priming task in which the age factor is emphasized (as in Experiment 1), rather than the emotional valence factor (as in Experiment 2).

## Materials and method

### Participants

Twenty-six undergraduate students (mean age = 24.1 years, SD = 1.8, range = 22–29), right-handed native Hebrew speakers, with normal or corrected-to-normal visual acuity, who did not participate in previous experiments, participated in Experiment 3 for payment. All were naïve regarding the purpose of the experiment. The sample size was based on a statistical power analysis using G*Power^27^ (see note 4 in Appendix B).

### Stimuli

Four images representing young children and four images representing young adults, all males, were chosen from the image pool used in Experiment 1. An additional four pictures representing old male adults were chosen from the IAPS database^28^. A pilot of eight independent participants judged the age of these old adults as 56–80 years old (mean = 70.25, SD = 4.9). All pictures included a single figure and were characterized by medium levels of valence and arousal, as indicated by IAPS (mean valence: for the young children category—5.4, SD = 1.6; adult category—5.3, SD = 1.3; old category—5.0, SD = 1.1; mean arousal: for the children—3.6, SD = 1.9; adult—3.6, SD = 2.1; old—3.1, SD = 1.7; see Table A.3 in Appendix A). Importantly, in contrast to Experiment 2, none of the figures within the image stimuli were characterized by a clear emotional expression (e.g., smile, crying), rather, they all depicted rather neutral facial expressions. Note that despite the similar explicit valence and arousal ratings, as indexed by the IAPS database, we hypothesized that implicit and possibly unconscious stereotypical attitudes towards elders would modulate responses in the numeral-classification task. Indeed, it has been shown that during the integration process of perception, “hidden” social category activations are often triggered, which temporarily impact perception without necessarily manifesting in explicit perceptual judgments^34^. With regard to old adults, Levy^35^ has specifically suggested that aging stereotypes can operate “below awareness” (p. 203). We therefore hypothesized that the parity judgment task conducted on the target digits may reveal implicit attitudes that were not reflected by the IAPS explicit valence ratings.

Images were presented on the screen as in Experiment 1. Target stimuli either depicted small (1 or 2), medium (4 or 5), or big (8 or 9) numerical values. Each of the four prime images within the three age conditions was presented with each of the two digits within the three number conditions, and each of these combinations was repeated twice during the block, yielding a total of 144 trials per block. Participants conducted 36 practice trials to get acquainted with all priming pictures before performing the two experimental blocks (yielding an overall total of 288 experimental trials).

### Procedure

The procedure followed that of Experiment 1B, in which a parity judgment task was performed, with one addition: Participants were instructed to respond aloud to the prime image by indicating whether the person in the image was “Young” (i.e., a child), an “Adult”, or “Old” (i.e., elderly adult). Note that in Hebrew all three terms are single-word nouns (with no verbal overlap between terms, such as in the case of “adult” and “old-adult” in English). All other procedure parameters were identical to those in Experiment 1B.

## Results

Accuracy and mean RTs for correct responses were computed for each participant in each condition. Outlier responses (i.e., responses that were shorter than 150 ms or were three standard deviations above/below the mean of each participant within each condition) were excluded from the analysis (1.2%). The overall error rate was very low (0.03), thus errors were not analyzed.

**Figure 4:**
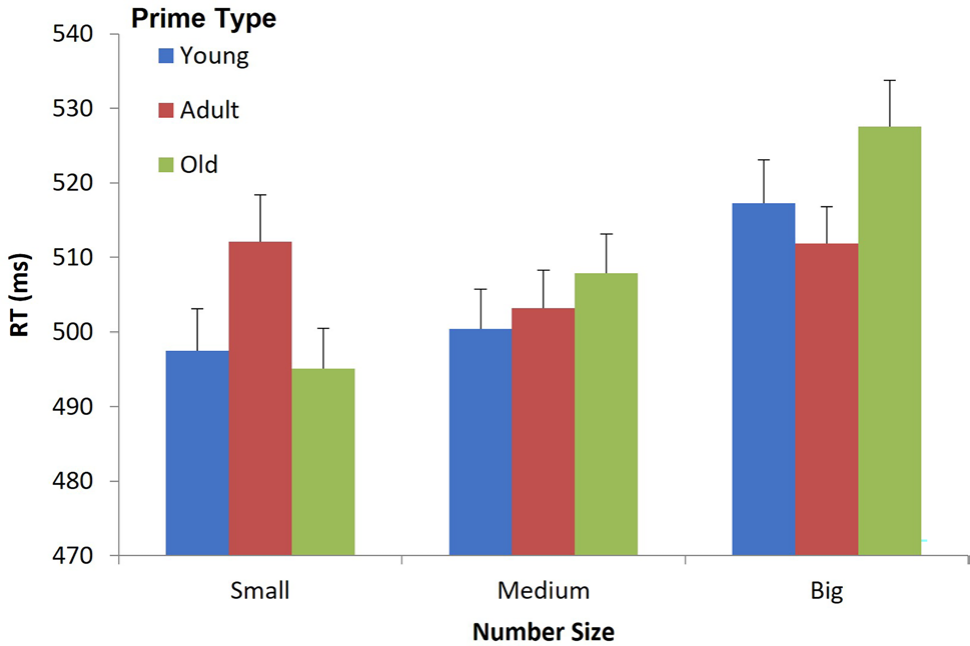
Mean reaction times (RT) to the parity-judgment task, within the different conditions (Prime type, Number size) in Experiment 3. Error bars indicate the standard errors of the means.

A repeated-measures ANOVA was conducted with Prime type (young, adult, old) and Number size (small, medium, big) as factors. A main effect for Number size was significant, F(2, 50) = 7.97, $\upeta_{{\text{p}}}^{2}$ = 0.24, MSE = 862, *p* > 0.0009. No main effect was found for Prime type, F(2, 50) = 1.1, ns. The two-way interaction between Number size and Prime type was significant, F(4, 100) = 2.94, $\upeta_{{\text{p}}}^{2}$ = 0.105, MSE = 528, *p* > 0.023. We replicated previous findings, showing faster RTs when images of young children preceded small numbers (1–2), compared to images of young adults, F(1, 25) = 6.8, MSE = 380, *p* > 0.015. When adult pictures preceded big numbers (8–9), faster RTs were observed, compared to young prime pictures, but this effect did not reach statistical significance, F(1, 25) < 1. More importantly, as hypothesized, the relation between age and number size was not linear when the old adult images came into play: rather than eliciting long RTs when preceding small numbers, old adult images elicited shorter RTs compared to the young adult prime images*,* F(1, 25) = 5.62*,* MSE = 631, *p* > 0.025; and, similarly, they elicited numerically longer RTs than the young adult images when preceding big numbers, though this difference was non-significant, F(1, 25) = 1.99, MSE = 1099, *p* > 0.17 (see Fig. 4).

These results, demonstrating an associative relation of old adults to small rather than to big numbers, clearly suggest that social stereotypes and emotional valence factors have overpowered the effects of objective age judgment. In fact, old adults’ prime images evoked target-related responses that resembled (or even exceeded) the responses associated with the young child prime images (see leftmost and rightmost graphs in Fig. 4, referring to the two extreme number categories). This finding has two important implications. First, it suggests that old adults are associated with a diminished or an inferior magnitude representation, which resembles that of young children. Thus, in contrast to a potential linear age representation, old adults are not perceived as “large”, but rather as “small”. Second, it provides important evidence against a pure “objective” magnitude explanation for the results, accounted for by conceptual size representation. As mentioned in Experiment 1 (where only child and young adult prime images were used), age and real-world size are largely correlated, since young adults are both older and physically larger than children. Due to this confound between the two objective magnitudes, conceived size, rather than age, may have accounted for the results of Experiment 1. However, this could not have been the case in the current experiment. Although old adults may be shorter (and/or more stooped) than young adults, they are clearly taller (and wider) than young children. Namely, the current pattern of results cannot be accounted for by real-world physical size factors, further suggesting a central role for stereotypical attitudes in old adults’ representation.

## General discussion

In the present study, we examined the potential interaction between an objective representation of human age and a more subjective, stereotypical percept associated with aging, through their joint influence on numerical size perception. Using a priming paradigm, we first established the associative (apparently linear) relation between age and numeral value representations. Then, we demonstrated an associative linkage of emotional valence to number perception (where negative images depicting weakness and/or poorness were associated with small numeral magnitudes while positive images were associated with big magnitudes), in accordance with Holmes and Lourenco^27^. Finally, we showed that when subjective social stereotypes associated with negative percepts potentially compete with objective age representation, the former dominate the latter in their influence on numerical size perception.

These findings deepen our understanding of objective magnitude perception, and its vulnerability to subjective biases, which stem from emotional valences evoked by the judged stimuli and/or from social attitudes associated with them. As mentioned earlier, several studies have already shown subjective biases influencing judgments regarding objects’ physical size. For instance, Bruner and Goodman^22^ showed that children with low socio-economic status, but not children with high socio-economic status, overestimated the size of a coin compared to the size of a gray disc of identical size. The greater the value of the coins, the greater was the deviation of apparent size from the actual size, suggesting a positive relation between size estimation and a desired value (see also^23^). Other studies showing a similar pattern of results were accounted for by a token-value hypothesis, suggesting that objects seen as valuable and/or powerful are overestimated^36^. An association between social power and physical size was also demonstrated by Schubert, Waldzus, and Giessner^37^, who observed that participants responded faster when the size of a label’s font matched the perceived power inferred by the label (e.g., “Professor” written in large font), than when it conflicted with it (“Student” written in a large font). Here we focused on real-world images of different age categories and their associative relations to numerical size. Our results demonstrate that old adults are associated with small numerical values, relative to young adults, presumably due to negative stereotypical percepts (such as weakness and vulnerability) associated with the former. This association was revealed even though prime images were strictly irrelevant (i.e., orthogonal) to the task completed on the digit targets, further emphasizing the automatic nature of this association. Numerical age is objectively linear, and spacing age groups in this study was made in an attempt to keep this linearity. Yet, it is possible that perception of age was not equally spaced among all participants. Nevertheless, the data suggest that something other than objective age contributes to priming. Eliminating physical size, valence and arousal, which should have placed older adults closer to young adults and not to children, the subjective stereotype perception receives more strength.

From a computational format of the representation of stereotypes, bearing both the parsimonious nature of our system and the competing routes that often operate during processing, we may offer the following framework for understanding the saliency of ageism in ones' mind: Stereotypes are pieces of information that are loaded by various parameters. Some are objective, such as chronological age; and some are subjective, such as feelings of aversion, death anxiety etc. The more a piece of information is loaded with subjective aspects, the more weight it will gain and hence, be more powerful in affecting a decision. In addition, the more emotional the subjective load, the more weight the information gains. This line of calculation makes it understandable that negative stereotypes dominate other information relevant for decision making, even when the stimulus is not central to the task. This is a very initial rational that should clearly receive further research and conceptualization.

Our results point to the social magnitude perception that accompanies age. Our categorization task for prime images, requesting participants to name whether the prime image presented a young/old person may have evoked objective magnitude percepts (since the definition of old is related to objective age). Yet, the social perception apparently emerged without explicit information. It would be interesting for future studies to further explore the emergence of social concepts of old age, even with non-magnitude related instructions. This could be done, for example, using a categorization task such as gender or hair color, just to ensure processing of the prime images.

A major limitation of our study relates to the prime stimuli used in the critical Experiment 3. Although IAPS images captured different age categories (children vs. young adults vs. old adults), they were not controlled well enough for other factors. The “old” images were lower in overall valence and arousal than both the “young children” and “adult” images (see Table A.3 in Appendix A). These differences were not statistically significant but nevertheless may raise the option that age is possibly partially confounded with the inherent valence and arousal of the stimuli. This limitation should receive further examination.

The importance of developing implicit measures for ageism is known^38^. Here, our results revealed that old adults are perceived as smaller than, and presumably inferior to, young adults. This concept appears to affect judgment, even when irrelevant, pointing to its’ saliency. To allow this interpretation, we conducted our experiments gradually, first establishing the independent connections between age and numerical magnitude, and valence and numerical magnitude, and then showing the combined effects that are inherent in the old adult concept. Further research is needed to broaden our understanding of the influential interactions between different social concepts and their effects on old adult representation. For instance, it would be interesting to examine the implicit attitudes towards weak/vulnerable young adults versus strong/powerful old adults, by matching each of the age categories with a powerful/powerless profession (e.g., a court judge vs. a street cleaner, respectively). Furthermore, while the present study was conducted among young students, it is important to explore implicit percepts of aging among populations of old adults, as these may differ significantly from the ones of young adults, and within themselves (e.g., old adults who possess negative self-stereotypes vs. old adults who possess positive self-stereotypes)^35^ and compare implicit findings to explicit reports, as a convergent validity examination. Future research will directly examine these questions.

### Supplementary Information

Supplementary Information 1.

Supplementary Information 2.

## Data Availability

The datasets generated and/or analyzed in the current study are available either in the Supplementary Materials or from the corresponding author on reasonable request.
