# Supplementary material for: Observing ageism implicitly using the numerical parity judgment task
Source: Sci Rep. 2023 Dec 1;13:21195. doi: 10.1038/s41598-023-40876-1 (PMC10692192; doi:10.1038/s41598-023-40876-1)
Supplement: Supplementary file 1 — Supplementary Information 1. [file 41598_2023_40876_MOESM1_ESM.docx]

**Appendix A (Tables)**

**Table A.1**

Mean and standard deviation (SD) of valence and arousal parameters for prime pictures used in Experiments 1A and 1B, taken from the IAPS (Lang et al., 2008).

| Young children images | | | | | |
| --- | --- | --- | --- | --- | --- |
|  | Slide No. | Valence - Mean | Valence - SD | Arousal - Mean | Arousal - SD |
| 1 | 2270 | 6.28 | 1.62 | 3.15 | 2.03 |
| 2 | 2795 | 3.92 | 1.77 | 4.7 | 2 |
| 3 | 9070 | 5.01 | 1.89 | 3.63 | 2.03 |
| 4 | 2440 | 4.49 | 1.03 | 2.63 | 1.7 |
| 5 | 2280 | 4.22 | 1.54 | 3.77 | 1.89 |
| 6 | 2442 | 6.17 | 1.4 | 4.04 | 1.89 |
| Average | | 5.015 | 1.541667 | 3.653333 | 1.923333 |
| Adult images | | | | | |
|  | Slide No. | Valence - Mean | Valence - SD | Arousal - Mean | Arousal - SD |
| 1 | 2215 | 4.63 | 1.24 | 3.38 | 2 |
| 2 | 2104 | 4.42 | 1.09 | 3.11 | 1.84 |
| 3 | 2107 | 5.53 | 1.05 | 3.72 | 2.12 |
| 4 | 4571 | 5.49 | 1.52 | 3.54 | 2.31 |
| 5 | 2493 | 4.82 | 1.27 | 3.34 | 2.1 |
| 6 | 8320 | 6.24 | 1.78 | 4.27 | 2.21 |
| Average | | 5.188333 | 1.325 | 3.56 | 2.096667 |

**Table A.2**

Mean and standard deviation (SD) of valence and arousal parameters for prime pictures used in Experiments 2A and 2B, taken from the IAPS (Lang et al., 2008).

| Negative images | | | | | |
| --- | --- | --- | --- | --- | --- |
|  | Slide No. | Valence - Mean | Valence - SD | Arousal - Mean | Arousal - SD |
| 1 | 2457 | 3.2 | 1.51 | 4.94 | 2.01 |
| 2 | 2301 | 2.78 | 1.38 | 4.57 | 1.96 |
| 3 | 2375.1 | 2.2 | 1.31 | 4.88 | 2.21 |
| 4 | 2750 | 2.56 | 1.32 | 4.31 | 1.81 |
| 5 | 3300 | 2.74 | 1.56 | 4.55 | 2.06 |
| Average | | 2.696 | 1.416 | 4.65 | 2.01 |
| Positive images | | | | | |
|  | Slide No. | Valence - Mean | Valence - SD | Arousal - Mean | Arousal - SD |
| 1 | 2260 | 8.06 | 1.42 | 4.26 | 2.44 |
| 2 | 2306 | 7.08 | 1.37 | 4.46 | 2.11 |
| 3 | 2030 | 6.71 | 1.73 | 4.54 | 2.37 |
| 4 | 8120 | 7.09 | 1.36 | 4.85 | 2.13 |
| 5 | 2040 | 8.17 | 1.6 | 4.64 | 2.54 |
| Average | | 7.422 | 1.496 | 4.55 | 2.318 |

**Table A.3**

Mean and standard deviation (SD) of valence and arousal parameters for prime pictures used in Experiment 3, taken from the IAPS (Lang et al., 2008).

| Young children images | | | | | |
| --- | --- | --- | --- | --- | --- |
|  | Slide No. | Valence - Mean | Valence - SD | Arousal - Mean | Arousal - SD |
| 1 | 2270 | 6.28 | 1.62 | 3.15 | 2.03 |
| 2 | 9070 | 5.01 | 1.89 | 3.63 | 2.03 |
| 3 | 2280 | 4.22 | 1.54 | 3.77 | 1.89 |
| 4 | 2442 | 6.17 | 1.4 | 4.04 | 1.89 |
| Average | | 5.42 | 1.6125 | 3.6475 | 1.96 |
| Adult images | | | | | |
|  | Slide No. | Valence - Mean | Valence - SD | Arousal - Mean | Arousal - SD |
| 1 | 2215 | 4.63 | 1.24 | 3.38 | 2 |
| 2 | 2107 | 5.53 | 1.05 | 3.72 | 2.12 |
| 3 | 2493 | 4.82 | 1.27 | 3.34 | 2.1 |
| 4 | 8320 | 6.24 | 1.78 | 4.27 | 2.21 |
| Average | | 5.305 | 1.335 | 3.6775 | 2.1075 |
| Old images | | | | | |
|  | Slide No. | Valence - Mean | Valence - SD | Arousal - Mean | Arousal - SD |
| 1 | 2495 | 5.22 | 1.1 | 3.19 | 1.76 |
| 2 | 2499 | 5.34 | 1.43 | 3.08 | 1.73 |
| 3 | 2570 | 4.78 | 1.24 | 2.76 | 1.92 |
| 4 | 2512 | 4.86 | 0.84 | 3.46 | 1.75 |
| Average | | 5.05 | 1.1525 | 3.1225 | 1.79 |
