# Supplementary material for: Observing ageism implicitly using the numerical parity judgment task
Source: Sci Rep. 2023 Dec 1;13:21195. doi: 10.1038/s41598-023-40876-1 (PMC10692192; doi:10.1038/s41598-023-40876-1)
Supplement: Supplementary file 2 — Supplementary Information 2. [file 41598_2023_40876_MOESM2_ESM.docx]

**Appendix B (Notes)**

1The type of power analysis was determined a priori, given a repeated-measures ANOVA, with within factors: an expected effect size of 0.5 for the two-way interaction between Prime type and Number value, considering α error probability = .05 and a satisfactory power 1-β error probability = 0.95, in each group. This yielded an N value of 17. In order to balance between response key pressings, an even number of participants was required, thus, 18 participants were recruited altogether.

2 In Hebrew both terms are typically used as nouns. The word “young” is comparable in many aspects to the word “adult” and therefore it was chosen to describe the children’s images. Other verbal alternatives to the word “young” (such as “child”) are used more frequently and are acquired in an earlier developmental stage than the word “adult” (see Henik, Rubinstein & Anaki, 2005: Henik, A., Rubinstein, O. & Anaki, D. *Word norms for the Hebrew language* (Ben Gurion University of the Negev, 2005).

3 Our study investigated negative valance associated with elder stereotypes, therefore, we focused on affects associated with percepts of poorness, weakness and/or vulnerability (versus competence and/or strength). As mentioned above, studies investigating size perception among poor or weak populations (e.g., children from a low socio-economic background) have shown a positive relation between the desirability of a stimulus and its perceived size. Namely, stimuli conceived as positive/desired were perceptually accentuated and were perceived as larger than neutral stimuli (e.g., Bruner & Goodman, 1947; Smith, Fuller, & Forrest, 1975). Other studies, however, have shown that *negative* stimuli related to threat or to aversion, and associated with feelings of fear or disgust, are actually linked to large size magnitudes, relative to non-threatening stimuli (e.g., Leibovich, Cohen, & Henik, 2016; van Ulzen, Semin, Oudejans, & Beek, 2008; Whitehouse, Freeman, & Annandale, 1988). While these two classes of findings may be seen at first to be contradictive, we believe that they reflect a true dissociation in the representation of different types of negative emotional valances: the former (i.e., sadness/incompetence) are associated with small magnitude representations, while the latter (i.e., fear/disgust) are associated with large magnitude representations. Clearly, further research is required to validate this proposed dissociation and its relations to magnitude perception. For the purposes of the current study, however, due to the investigation of negative stereotypes associated with elders, we focus on feelings evoked by images depicting poorness and/or sadness only.

Leibovich, T., Cohen, N. & Henik, A. Itsy bitsy spider? Valence and self-relevance predict size estimation. *Biol. Psychol.* **121**, 138-145 (2016).

Van Ulzen, N. R., Semin, G. R., Oudejans, R. R. & Beek, P. J. Affective stimulus properties influence size perception and the Ebbinghaus illusion. *Psychol. Res.* **72**, 304-310 (2008).

Whitehouse, A. M., Freeman, C. P. & Annandale, A. Body size estimation in anorexia nervosa. *Br. J. Psychiatry* **153**, 23–26 (1988).

4 The type of power analysis was a priori, given a repeated-measures ANOVA, with within factors: an expected effect size of 0.22 for the two-way interaction between Prime type and Number type, considering α error probability = .05 and a satisfactory power 1-β error probability = 0.95, in one group. This yielded an N of 25. In order to balance between response key pressings, an even number of participants was required, thus, 26 participants were recruited.
